# Supplementary figures and images for: Gαq and Phospholipase Cβ signaling regulate nociceptor sensitivity in Drosophila melanogaster larvae
Source: PeerJ. 2018 Sep 20;6:e5632. doi: 10.7717/peerj.5632 (PMC6151255; doi:10.7717/peerj.5632)

# Microarray detection of mdIV transcripts

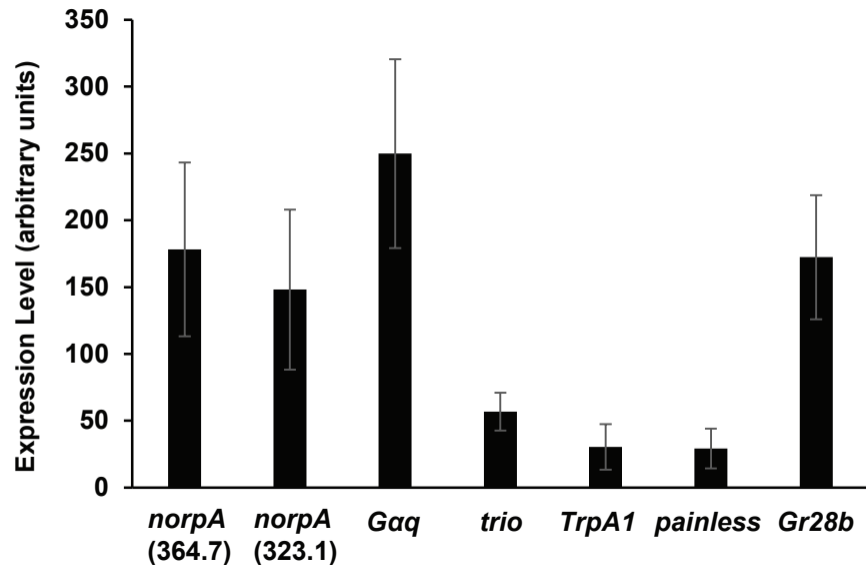

Supplement: Figure S1 — Normalized detection values were taken from a published microarray dataset (ArrayExpress Accession # E-MTAB-3863) produced using the GeneChip Drosophila Genome 2.0 Array. The dataset contains four biological replicates, each consisting of 40–50 mdIV neuron cell bodies collected via laser capture microdissection. Each bar indicates the mean normalized detection value for four biological replicates. Error bars indicate standard error. [file peerj-06-5632-s001.pdf]
